# Evaluation of Antiproliferative Activity of Red Sorghum Bran Anthocyanin on a Human Breast Cancer Cell Line (MCF-7)

**DOI:** 10.4061/2011/891481

**Published:** 2011-10-16

**Authors:** P. Suganya Devi, M. Saravana Kumar, S. Mohan Das

**Affiliations:** ^1^P.G. Department of Biotechnology, Dr. Mahalingam Centre for Research and Development, NGM College, Pollachi 642001, India; ^2^Kamadhenu Arts and Science College, Sathyamangalam 638503, India

## Abstract

Breast cancer is a leading cause of death in women worldwide both in the developed and developing countries. Thus effective treatment of breast cancer with potential antitumour drugs is important. In this paper, human breast cancer cell line MCF-7 has been employed to evaluate the antiproliferative activity of red sorghum bran anthocyanin. The present investigation showed that red sorghum bran anthocyanin induced growth inhibition of MCF-7 cells at significant level. The growth inhibition is dose dependent and irreversible in nature. When MCF-7 cells were treated with red sorghum bran anthocyanins due to activity of anthocyanin morphological changes were observed. The morphological changes were identified through the formation of apoptopic bodies. The fragmentation by these anthocyanins on DNA to oligonuleosomal-sized fragments, is a characteristic of apoptosis, and it was observed as concentration-dependent. Thus, this paper clearly demonstrates that human breast cancer cell MCF-7 is highly responsive by red sorghum bran anthocyanins result from the induction of apoptosis in MCF-7 cells.

## 1. Introduction

In India among the urban women, the numbers of breast cancer patients were increasing annually, both to aging of the population and increase in age-specific incidences [1]. Case control studies in Mumbai and Chennai have identified the factors such as null parity, late age at marriage, and late age at first pregnancy are important risk factors. [2, 3]. It has also been suggested that western dietary influences changed the lifestyle of urban women could be one of the major causes of the slowly rising incidence of breast cancer in India [1]. Therefore early detection and search for potential antitumour compounds are important in the control of breast cancer.

We have extracted anthocyanins from red sorghum bran by methanol and acidified methanol and evaluated the antitumour activity in MCF-7 cell lines. The efficiency of the antitumour compounds seems to be related to the propensity of tumour cells to respond to these sorghum anthocaynins by apoptosis. Recently, considerable attention has been focused on the sequence of events referred to as apoptosis and the role of this process in mediating the lethal effects of antineoplastic agents in breast cancer cells. Apoptosis is a highly regulated process that is characterized by cell shrinkage, membrane blebbing, chromatin condensation and formation of a DNA ladder with multiple fragments of 180–200 bp caused by internucleosomal DNA cleavage [4]. Few recent studies of anthocyanins have demonstrated a significant growth inhibition of some tumour cells including human colon cancer, human cervical carcinoma, human leukaemia, and prostate cancer cells [5–8].

Considering the antiproliferative activity of some anthocyanins on certain neoplastic cells, an attempt has been made to evaluate the growth inhibitory activity of a relatively sorghum anthocyanins on MCF-7 cells, and that may provide some new information about therapy of breast cancer. 

## 2. Materials and Methods

### 2.1. Samples

The bran of *Sorghum bicolor *(L.) red sorghum were collected from farmers field in Tamil Nadu, India and were stored at −20°C.

### 2.2. Anthocyanin Extraction

The anthocyanin extraction protocol involved the addition of 10 ml of solvent (1% HCl in methanol) to 0.5 g of sample in 50 ml centrifuge tubes and shaking the samples for 2 hours at low speed (75 rpm) in an orbitory shaker (Neolab). Samples were then stored at −20°C for overnight in the dark to allow for maximum diffusion of phenolics from the cellular matrix. Samples were then equilibrated to room temperature and centrifuged at 7000 rpm for 10 min and taken for analysis. Residues were rinsed with 10 ml volumes of solvent for two times with shaking for 5 min, then centrifuging at 7000 rpm for 10 min and taken for analysis. Finally, the extracts were mixed well and stored at −20°C in the dark until further biochemical analysis [9].

### 2.3. Cell Culture

Human breast cancer cell line, MCF-7, cells were maintained in DMEM containing 10% FBS, supplemented with additional glutamine (0.03%) and 100 *μ*g/mL benzyl penicillin, 100 U/mL streptomycin, and 2.5 *μ*g/mL amphotericin. Cells were allowed to grow in tissue culture flasks (Corning, USA) and were kept in CO_2_ incubator at 37°C in a humidified atmosphere of 5% CO_2_ and 95% air. For experimental purpose, cells from exponentially growing culture were used. All experiments were repeated three times.

### 2.4. Cell Viability Assay

Cell viability assays were carried out as described by [10]. Briefly, cells were seeded at a density of 3–104 cells/well into 24-well plates. After 24 h, GA extracts were added to the medium at various concentrations and incubated for 24 or 48 h as indicated. At the end of the incubation, 3-(4-5 dimethylthiozol-2-yl), 2–5-diphenyl-tetrazolium bromide (MTT) (2 mg/mL) per well was added, and the formazan crystals formed were solubilized in acidified isopropanol after aspirating the medium. The extent of MTT reduction was measured spectrophotometrically at 570 nm, and the cell survival was expressed as percentage over the untreated control. 

### 2.5. Cell Morphology Studies

The methanol and acidified methanol extracts of sorghum bran on cell morphology of MCF-7 cells was investigated. MCF-7 cells grown in a 6-well plates were treated with different extracts of anthocyanin at 37°C for 24 h. Morphological changes occurring in the cells were observed under phase-contrast microscope and photographed using CCD camera attached to the TE 2000 E microscope (Nikon).

### 2.6. DNA Fragmentation by Agarose Gel Electrophoresis

Nuclear morphology changes characteristic of apoptosis appear within the cell together with a distinctive biochemical event: the endonuclease-mediated cleavage of nuclear DNA. In fact, formation of DNA fragments of oligonucleosomal size (180–200 bp) is an hallmark of apoptosis in many cell types. 

The present protocol provides a method for DNA separation of fragmented and intact DNA fractions and for their analysis by agarose gel electrophoresis. In apoptotic cells specific DNA cleavage becomes evident in electrophoresis analysis as a typical ladder pattern due to multiple DNA fragments. However, although this protocol is simple and generally able to provide good results, it is only qualitative because of its limitations in DNA recovery and solubilization. In order to obtain a cleaner DNA, other methods for DNA preparation are required (in some cases use of proteinase K for deproteinization is recommended).

#### 2.6.1. Materials

Cell suspension at 1–5 ×10^6^ cells/mL in complete MEM medium TTE solution: TE buffer pH 7.4 with 0.2% Triton X-100 (store at 4°C) NaCl 5 M, ice-cold isopropanol, ice-cold ethanol at 70%, ice cold TE buffer pH 7.4 loading buffer 10x TBE buffer for electrophoresis ethidium bromide solution electrophoresis-grade agarose DNA molecular weight markers refrigerated cell centrifuge gel electrophoresis apparatus UV transilluminator. 

#### 2.6.2. Methodology

1. Dispense 0.5 mL of cell suspension (no less than 5 × 10^5^, otherwise DNA will not be detectable by photography of ethidium bromide stained gel, and no more than 5 × 10^6^, to avoid difficult handling of too high amounts of insoluble DNA) in tubes labeled B (bottom).
2. Centrifuge cells at 200 ×g at 4°C for 10 min.
3. Transfer supernatants carefully in new tubes labeled S (supernatant).
4. Add to the pellet in tubes B 0.5 mL of TTE solution and vortex vigorously. This procedure allows the release of fragmented chromatin from nuclei, after cell lysis (due to the presence of Triton X-100 in the TTE solution) and disruption of the nuclear structure (following Mg^++^ chelation by EDTA in the TTE solution).
5. To separate fragmented DNA from intact chromatin, centrifuge tubes B at 20,000 ×g for 10 min at 4°C.
6. Carefully transfer supernatants in new tubes labeled T (top).
7. Add to the small pellet in tubes B 0.5 mL of TTE solution.
8. Add to the 0.5 mL volume present in tubes B, S and T, 0.1 mL of ice-cold 5 M NaCl and vortex vigorously. The addition of the salt should be able to remove histons from DNA.
9. Add to each tube 0.7 mL of ice-cold isopropanol and vortex vigorously.
10. Allow precipitation to proceed overnight at −20°C. This step can be shortened by putting samples in a bath of ethanol/dry ice for 1 hr.
11. After precipitation, recover DNA by pelleting for 10 min at 20,000 ×g at 4°C.
12. Discard supernatants by aspiration or by rapidly inverting tubes and carefully remove any drops or fluid remaining adherent to the wall of the tube with a paper towel corner. This can be a critical step because the pellet could be loosened and transparent, hard to be seen.
13. Rinse the pellets by adding to each tube 0.5–0.7 mL of ice-cold 70% ethanol.
14. Centrifuge tubes at 20,000 ×g for 10 min at 4°C.
15. Discard supernatants by aspiration or by rapidly inverting tubes. Carefully remove any drops or fluid remaining adherent to the wall of the tube by inverting tubes over an absorbent paper towel for 30 min. Let air dry the tubes in upright position for at least 3 hr before proceeding.
16. Dissolve DNA by adding to each tube 20–50 l of TE solution and place the tubes at 37°C for 1–3 days. The redissolution of DNA may be a critical step, in fact it depends on the DNA quantity and size present in the samples. Thus, the nonfragmented DNA contained in the B tubes, may need higher volumes of TE and longer incubation times in order to be resuspended.
17. Mix the samples of DNA with loading buffer by adding 10x loading buffer to a final concentration of 1x. The addition of loading buffer to samples allows to load gel wells more easily and to monitor the run of samples.
18. Place samples in a heating block at 65°C for 10 min and immediately load 10–20 l of them to each well of a standard 1% agarose gel containing ethidium bromide 0.5 mg/mL. Appropriate DNA molecular weight markers should be included. Ethidium bromide is a potential carcinogen: wear gloves and handle with care.
19. Run the electrophoresis in standard TBE buffer after setting the voltage to the desired level. During electrophoresis it is possible to monitor the migration of samples by following the migration of bromophenol blue dye contained in the loading buffer.
20. Stop the electrophoresis when the dye reaches about 3 cm from the end of the gel.
21. To visualize DNA, place the gel on a UV transilluminator and take photos of the gel. Wear eye and skin protection when UV is on.

## 3. Results

### 3.1. Morphological Changes

**Figure 1:**
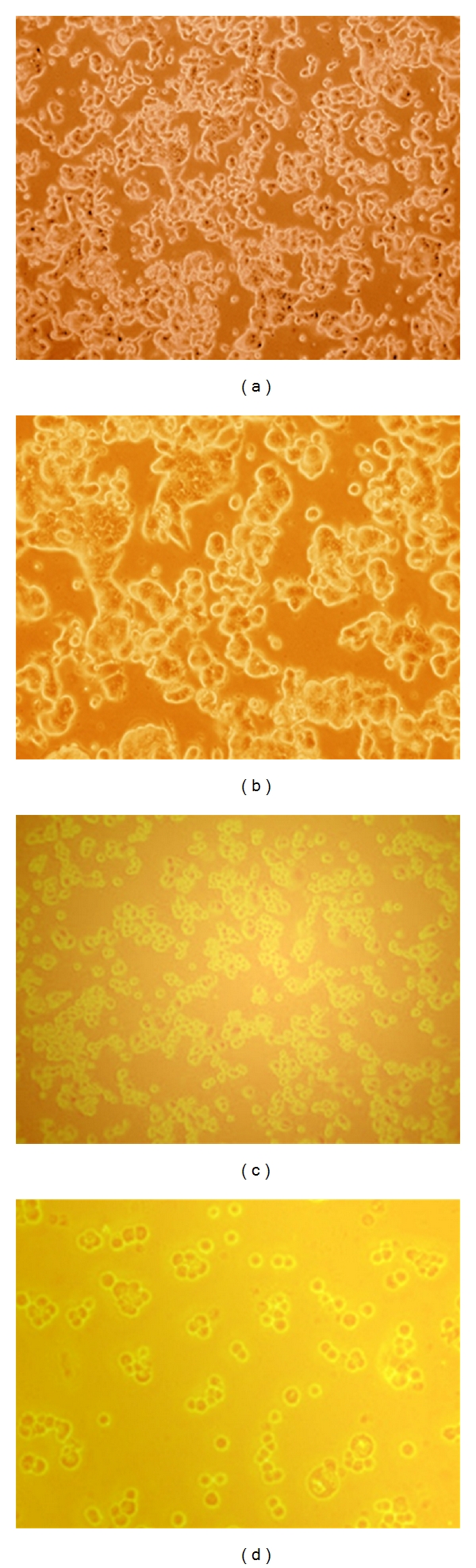
(a) Normal MCF-7 cells. (b) Anthocyanin treated MCF-7 cells showing spherical shaped cells leading to loss of cell anchorage with concentration of 1000 *μ*g/mL. (c) Anthocyanin treated MCF-7 cells showing spherical shaped cells leading to loss of cell anchorage with concentration of 500 *μ*g/mL. (d) Anthocyanin treated MCF-7 cells showing spherical shaped cells leading to loss of cell anchorage and cell number was also reduced with concentration of 250 *μ*g/mL.

In the present study, antiproliferative effect of red sorghum bran anthocyanin on breast cancer cell line, MCF-7 was investigated under different concentrations. The red sorghum bran anthocyanins produced significant morphological alterations on MCF-7 cells in culture. Under normal growth condition (control) these cells were regular in shape and size, had eccentric nucleus and a relatively small piece of cytoplasm (Figure 1(a)). After treatment with 1000 *μ*g/mL of anthocyanin, cells became irregular in shape and size with altered nuclear: cytoplasm ratio, increased number of nucleoli and multiple cytoplasmic vacuoles (Figure 1(b)). Most of the cells had relatively flattened appearance with long multiple cytoplasmic processes forming cross-bridges with neighboring cells (Figure 1(c)). This has indicated that anthocyanins may render some changes on the cell surface associated with the adherence to the substratum, as a result of which, treated cells tend to adhere firmly to the growth surface, an opposite behavior of the tumor cells and *in vivo* systems [11]. After anthocyanin treatment appearance of multinucleated giant cells were also observed in MCF-7 cells (Figure 1(d)). 

### 3.2. Assay for Growth Inhibition

**Figure 2:**
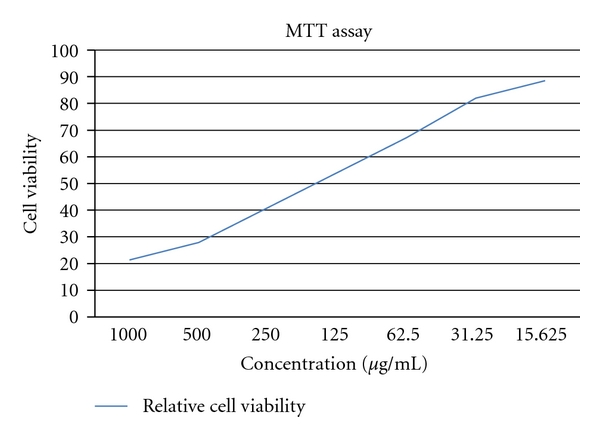
Relative cell viability of MCF-7 cells at different concentration of anthocyanin extracted by methanol.

The result demonstrate that, with increasing concentrations of sorghum anthocyanins from 15 *μ*g/mL to 1000 *μ*g/mL the percentage of growth inhibition was 21.31 in methanol extract after 24 hr of anthocyanin exposure. It was also evident that at all anthocyanin concentrations the percentage of growth inhibition increased with increase in concentrations (Table 1). Thus, the detailed analysis of the results clearly indicated that anthocyanins caused significant growth inhibition of MCF-7 cells in dose-dependent manner (Figure 2). 

**Figure 3:**
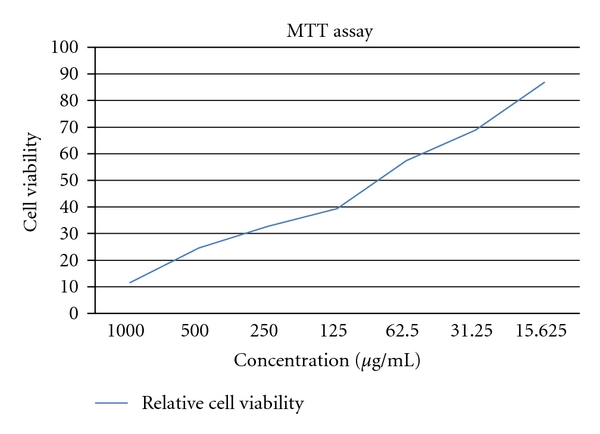
Relative cell viability of MCF-7 cells at different concentration of anthocaynin extracted by acidified methanol.

A similar result was observed when MCF-7 cells were treated with acidified methanol extracts of anthocyanin from sorghum bran. The result indicated that with increasing concentrations of sorghum anthocyanins from 15 *μ*g/mL to 1000 *μ*g/mL the percentage of growth inhibition of MCF-7 cells increased progressively from 11.47% to 86.88% in acidified methanol extract after 24 hr of anthocyanin exposure (Table 2), when compared with methanol, acidified methanol extract showed a higher growth inhibition (Figure 3).

Finally, we reported that the sorghum anthocyanin caused significant growth inhibition of human breast cancer cell lines even at 1000 *μ*g/mL concentration. No ealier reports are available on this aspects. Pouget et al. [12] and Han et al. [13] reported the anticancer activities of flavonoids like flavanones, daidzein, genistein, quercetin, and luteolin in human breast cancer by using MCF-7 cell line.

### 3.3. DNA Fragmentation

**Figure 4:**
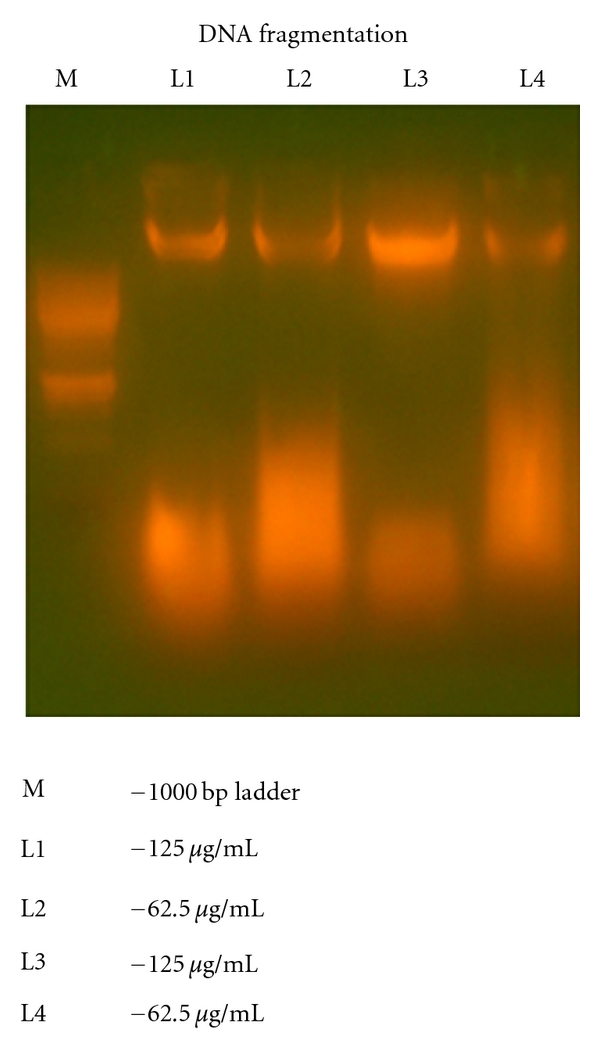
The DNA fragmentation analysis of oligonuceosomalsized fragments in the MCF-7 cells treated with red sorghum bran anthocyanins.

The significant growth inhibitory activities of red sorghum bran anthocyanins led us to investigate the effect of anthocyanin was also played a role in the induction of apoptosis. As shown in Figure 4, the amounts of oligonuceosomal-sized fragments in the MCF-7 cells treated with red sorghum bran anthocyanins were increased when the concentrations of these anthocyanins increased from 62.5 *μ*g/mL to 125 *μ*g/mL. Katsuzaki et al. [14] reported DNA fragmentation in leukemia cells molt 4B by cyanidin 3 glucoside isolated from skin of black *Glycine max.*

On the other hand, we have observed similar results in both methanol and acidified methanol extracts of red sorghum bran anthocyanin. Mukherjee et al. [15] evaluated the antiproliferative activity of enoxacin on a human breast cancer cell line which showed significant result after 5 days of drug exposure.

Correct the sentence: The present study demonstrated that these red sorghum bran anthocyanins have significant growth inhibitory effects on human breast cancer.

## 4. Conclusion

The above results clearly demonstrated that the anthocyanin has induced significant antiproliferative activity on breast cancer cells. Moreover the compound responsible for antiproliferative activity and the mechanism of action of these compounds are yet to be studied in detail. Further investigations in these aspects are required.

## Figures and Tables

**Table 1:** Showing the effect of anthocyanin on MCF-7 cells extracted from red sorghum bran by using methanol.

S. number	Concentration (*μ*g/mL)	Dilutions	Absorbance	Cell viability
1	1000	Neat	0.13	21.31
2	500	1 : 1	0.17	27.86
3	250	1 : 2	0.25	40.98
4	125	1 : 4	0.33	54.09
5	62.5	1 : 8	0.41	67.21
6	31.25	1 : 16	0.50	81.96
7	15.625	1 : 32	0.54	88.52
8	Cell control	—	0.61	100

**Table 2:** Showing the effect of anthocyanin on MCF-7 cells extracted from red sorghum bran by using acidified methanol.

S. number	Concentration (*μ*g/mL)	Dilutions	Absorbance	Cell viability
1	1000	Neat	0.07	11.47
2	500	1 : 1	0.15	24.59
3	250	1 : 2	0.20	32.78
4	125	1 : 4	0.24	39.34
5	62.5	1 : 8	0.35	57.37
6	31.25	1 : 16	0.42	68.85
7	15.625	1 : 32	0.53	86.88
8	Cell control	—	0.61	100
